# Morphology and composition play distinct and complementary roles in the tolerance of plantar skin to mechanical load

**DOI:** 10.1126/sciadv.aay0244

**Published:** 2019-10-09

**Authors:** Colin J. Boyle, Magdalena Plotczyk, Sergi Fayos Villalta, Sharad Patel, Shehan Hettiaratchy, Spyros D. Masouros, Marc A. Masen, Claire A. Higgins

**Affiliations:** 1 Department of Bioengineering, Imperial College London, London, UK.; 2 Department of Surgery, Imperial College London, London, UK.; 3 Department of Mechanical Engineering, Imperial College London, London, UK.

## Abstract

Plantar skin on the soles of the feet has a distinct morphology and composition that is thought to enhance its tolerance to mechanical loads, although the individual contributions of morphology and composition have never been quantified. Here, we combine multiscale mechanical testing and computational models of load bearing to quantify the mechanical environment of both plantar and nonplantar skin under load. We find that morphology and composition play distinct and complementary roles in plantar skin’s load tolerance. More specifically, the thick stratum corneum provides protection from stress-based injuries such as skin tears and blisters, while epidermal and dermal compositions provide protection from deformation-based injuries such as pressure ulcers. This work provides insights into the roles of skin morphology and composition more generally and will inform the design of engineered skin substitutes as well as the etiology of skin injury.

## Teaser

Skin morphology and composition play complementary roles in protecting plantar skin from stress- and deformation-induced injury.

## INTRODUCTION

Our skin continually bears mechanical loads as we interact with the environment around us. Excessive mechanical loads can lead to skin injuries such as pressure ulcers, age-associated skin tears, and blisters that have substantial consequences for human health. Pressure ulcers form during prolonged exposure to mechanical loads that are lower than those needed to rupture or physically damage the skin (*1*), for example, when patients interact with medical devices (*2*, *3*) and support surfaces (*4*). It is estimated that nearly one in five hospitalized patients develop pressure ulcers in European Union hospitals (*5*), with critically ill and neuropathic patients most at risk. Age-associated skin tears, which may be more prevalent than pressure ulcers, occur when acute loading causes rupture of the skin (*6*). Treating these collective skin injuries is estimated to cost the United Kingdom’s National Health Service £5 billion annually (*7*), while $50 billion is spent in the United States every year on treating chronic wounds resulting from skin injury (*8*).

One difficulty in preventing skin injury has been quantifying the load tolerance of skin. Many factors other than the magnitude and duration of loading affect an individual’s injury risk, such as their age, level of mobility, and degree of tissue perfusion (*9*). In addition, injury is more likely to occur under certain types of loading. For example, compression in combination with shear is more injurious than compression alone (*10*). Research over the past 20 years has sought to explain skin injury risk factors in terms of the local mechanical environment within tissues. Experimental models of pressure ulcers in which damage was induced in the hindlimbs of rats showed that the location of damaged tissue correlated with regions of high local deformations (*11*, *12*). Computational modeling of load-bearing soft tissue has shown that bony prominences induce substantial stress concentrations, which explains why these areas are vulnerable to ulceration (*13*, *14*). Recently, advances in computational modeling of skin under load have shown that stresses and strains are highly heterogeneous at the skin microstructural level (*15*). These computational models provide an opportunity to understand skin injury and identify the characteristics of load-tolerant skin.

Load tolerance varies considerably with anatomical location. Some anatomical locations, such as the pelvis, are highly vulnerable to injury (*5*), while the sole of the foot, known as the plantar region, is particularly resistant to load-induced injury. The plantar region has evolved to tolerate routine surface pressures of more than 1000 kPa (*16*), while skin at other load-bearing locations, such as the seated buttocks, rarely experience ^1^/_50_ of that magnitude (*17*). While some of the load-bearing properties of the foot can be attributed to specialized energy dissipation structures, such as the calcaneal and metatarsal fat pads (*18*), here, we hypothesize that the intrinsic properties of plantar skin are key features of the foot’s capacity to bear load and protect itself from mechanical injury. There are several observations that support this hypothesis. For example, when plantar skin is used as graft tissue on load-bearing sites, such as on plantar defects (*19*) or on the residual limbs of amputees (*20*), it can partially restore load-bearing function, while nonplantar skin grafts perform poorly on load-bearing sites (*21*). Furthermore, the back of the heel is highly vulnerable to injury (*5*) despite its proximity to the plantar region, indicating that load tolerance is highly localized to the sole of the foot. By comparing skin from load-tolerant and load-averse locations, we theorized that we would gain insights into the structural properties that enhance load tolerance.

**Fig. 1:**
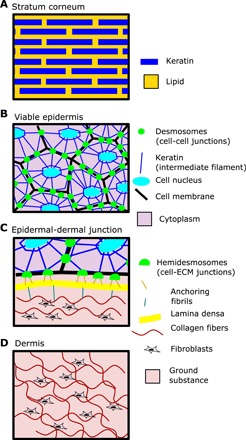
The load-bearing structures of skin. (**A**) The stratum corneum consists of terminally differentiated keratinocytes embedded in a lipid matrix and provides the contact surface for external mechanical loads. (**B**) In the viable epidermis, mechanical loads are borne directly by keratinocytes. Desmosomes provide mechanical junctions between neighboring cells, while keratin filaments provide the cell’s internal structural support. (**C**) The epidermal-dermal junction (EDJ) mechanically connects the dermis to the epidermis via the intermediate layer, the lamina densa. Anchoring fibrils connect the keratinocytes of the epidermis to the lamina densa (or basement membrane), and likewise, fibrils loop down from the lamina densa into the extracellular matrix (ECM) of the dermis. (**D**) Loads in the dermis are borne by extracellular matrix consisting of structural fibers, such as collagen and elastin, embedded in a ground substance consisting of glycosaminoglycans, proteoglycans, and water. Dermal fibroblasts create and maintain this matrix.

Skin has a complex load-bearing structure that varies considerably across its layers (Fig. 1). The skin on all body sites comprises a stratified epidermis and a vascularized dermis separated by a basement membrane (*22*). In the dermis, load is borne by extracellular matrix composed of fibers (primarily collagen and elastin) embedded in ground substance (primarily glycosaminoglycans, proteoglycans, and water). In the epidermis, load is borne by the cytoskeletons (primarily keratins) of epidermal cells that are joined together by cell-cell junctions (desmosomes). The outermost layer of the epidermis, the stratum corneum, consists of terminally differentiated keratinocytes that form a tough, impermeable barrier to the environment. The inner layers form the viable epidermis, where keratinocytes proliferate and differentiate to replenish the stratum corneum. The epidermis and dermis meet at the epidermal-dermal junction (EDJ), otherwise known as the basement membrane. Hemidesmosomes anchor the epidermal cells to the basement membrane, while a complex of proteins such as collagen VII loops from the basement membrane into the extracellular matrix of the dermis.

Plantar skin has a unique morphology and composition, and it is thought that these features enhance its load tolerance (*23*). In terms of morphology, the stratum corneum is much thicker in plantar skin than on other body sites, and there is greater interdigitation between the epidermis and dermis (*23*). In terms of composition, plantar epidermis contains the unique cytoskeletal protein keratin 9 (K9), the expression of which is regulated by a distal-specific Hox code expressed by fibroblasts in the skin dermis (*24*). This unique cytoskeletal profile is thought to enhance the load tolerance of palmoplantar skin (*23*), an idea reinforced by the observation that mutations in K9 lead to palmoplantar keratoderma, a skin disease characterized by hyperproliferation of sole and palm keratinocytes and skin blistering under loading conditions (*25*).

While the unique morphology and composition of plantar skin are well described, the extent to which each of these properties enhances plantar skin’s load tolerance has not been quantified. In this study, we set out to quantify how the morphology and composition of plantar skin enhance its tolerance to load. We quantified compositional differences between plantar and nonplantar skin using immunofluorescence imaging of structural proteins. We next quantified the mechanical response of both plantar and nonplantar skin using whole-skin and layer-specific mechanical testing. Last, we compared the effects of composition and morphology on load bearing using finite element computational modeling. We found that morphology and composition play distinct and complementary roles in enhancing plantar skin’s load tolerance. This research can be used to inform biomimetic engineering of skin substitutes for use on load-bearing body sites and provide targets to improve the load-bearing capacity of vulnerable skin, such as on the residual limb of an amputee.

## RESULTS

### Plantar skin has distinct morphology and composition

To understand plantar skin’s enhanced tolerance to load, we first quantified the morphological and compositional differences between plantar and nonplantar skin. We sectioned and imaged frozen samples of plantar and nonplantar human skin taken from the same patient. From these samples, we quantified the morphology of the epidermis, collagen structure in the dermis, and structural proteins in both the dermis and epidermis.

**Fig. 2:**
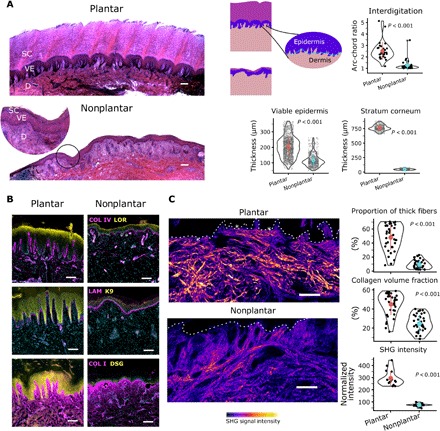
Morphology and composition of plantar skin. (**A**) H&E-stained sections indicating the morphological differences between plantar and nonplantar skin, including a thicker stratum corneum (SC) and viable epidermis (VE) in plantar skin (*n* = 414 and 1440 for stratum corneum and viable epidermis, respectively). There is greater interdigitation between the epidermis and dermis (D) in plantar skin as measured by the arc-chord ratio (*n* = 30 measurements). (**B**) Immunofluorescence imaging of skin sections shows that the dermis of plantar skin contains more capillaries [as indicated by collagen IV (COL IV) staining] compared to nonplantar skin. The epidermis of plantar skin uniquely expresses K9, while there is also higher expression of DSG1 in the epidermis and less LAM (laminin) and COL IV at the basement membrane. (**C**) SHG images showing collagen organization in the dermis reveal that the collagen in plantar skin is arranged in thicker bundles compared to nonplantar skin, and there are a greater proportion of thick fibers in plantar skin (*n* = 36 measurements). SHG signal intensity is significantly higher in plantar skin than in nonplantar skin (*n* = 14 measurements). Thick collagen fibers (asterisks) run parallel to the EDJ (dashed lines) in plantar skin. Scale bars, 200 μm. Reported *P* values are based on two-sided Student’s *t* tests.

To quantify differences in epidermal morphology, we stained skin sections using hematoxylin and eosin (H&E) and used image segmentation to define the boundaries of the viable (basal, spinous, and granular layers) and nonviable (stratum corneum layer) epidermis. We then measured two morphological characteristics: the thickness of the stratum corneum and the amount of interdigitation of the EDJ. Interdigitation was quantified by measuring the arc-chord ratio of the curve defining the EDJ. We found that the stratum corneum of plantar skin was 16 times thicker than the stratum corneum of nonplantar skin (Fig. 2A). We also found substantially greater interdigitation between the epidermis and dermis (2-fold higher median in plantar skin; Fig. 2A) of plantar skin.

We quantified differences in epidermal composition using immunofluorescence staining. The suprabasal epidermis of plantar skin expressed the cytoskeletal protein K9, whereas this was not present at detectable levels in nonplantar skin (Fig. 2B). Cells of the epidermis are mechanically linked through protein plaques in the cell membrane called desmosomes. Desmosomes have been shown to be critical to skin’s ability to bear tension under external loading (*26*). We quantified the expression of desmoglein 1 (DSG1; a key component of desmosomes) and found a 2.1-fold stronger fluorescence intensity in plantar skin compared to nonplantar skin. This higher DSG1 intensity could be due to either larger or more numerous desmosomes.

We quantified the differences in dermal composition using both second-harmonic generation (SHG) imaging and immunofluorescence imaging. Collagen I (COL1) is the main structural extracellular matrix protein in dermis, comprising approximately 90% of its dry weight. Collagen I fibers generate a second-harmonic light signal that can be used to visualize collagen structure in great detail using SHG microscopy. From SHG images of collagen, we used image segmentation to quantify the amount and thickness of collagen fibers in the dermis (fig. S1A). This analysis revealed not only that the volume fraction of collagen is greater in plantar dermis but also that there are a greater proportion of thick collagen fibers (defined as width > 10 μm) in plantar dermis.

The organization of collagen fibers in the dermis can also affect skin mechanics because collagenous tissue is stiffer when loaded parallel to the fiber direction (*27*). The SHG images revealed that thick collagen fibers penetrate close to the epidermis in plantar dermis, while in nonplantar dermis, collagen is present in finer, disorganized fibers (Fig. 2C). Collagen fibers in plantar dermis are preferentially oriented tangential to the epidermis, which is not the case for nonplantar dermis (fig. S1B). This network of thick fibers running parallel to the epidermis may protect plantar dermis from distortion under shear load.

The level of pretension on collagen fibers can affect skin mechanics. Collagen fibers provide strength by resisting tension, but fibers only begin to resist deformation when they become sufficiently stretched (*28*). Therefore, a tissue in which collagen is already tensed would be poised to resist deformation. To assess the level of pretension in dermal collagen, we quantified the SHG signal intensity, which has been used as a proxy measurement for tension in collagen fibers because more aligned fibers generate greater signals under polarized light (*29*). We found SHG signal intensity to be 4-fold greater in plantar skin samples, suggesting that plantar collagen fibers are under greater pretension than nonplantar fibers.

The mechanical connection between the epidermis and dermis depends on several structural proteins, including laminin and collagen IV. We hypothesized that these proteins would be more abundant in plantar skin than nonplantar skin to protect it from shear loads. Contrary to this hypothesis, we found that laminin and collagen IV fluorescence intensity at the EDJ was significantly lower in plantar skin compared to nonplantar skin (1.7- and 1.8-fold less laminin-332 and collagen IV, respectively; fig. S1C). The lower quantity of these basement membrane proteins suggests that the EDJ of plantar skin can bear less stress than that of nonplantar skin. Under this interpretation, the EDJ of plantar skin must be shielded from high stresses, rather than adapted to tolerate them. The epidermis is nutritionally supported by the dermis, so a less-dense basement membrane could also enable greater nutrient transfer from the dermis. Using collagen IV staining to mark capillaries, we found a 2.9-fold greater vessel density in plantar papillary dermis relative to nonplantar papillary dermis (Fig. 2B and fig. S1D), indicating a more nutritionally demanding tissue.

These results demonstrate that there are clear and measurable morphological and compositional differences between plantar and nonplantar skin. This raises key questions about the contribution of these differences to plantar skin’s tolerance to load. Does each of the differences found in plantar skin enhance its tolerance to load equally, or are some characteristics most important? Does the answer to this question depend on the mode of skin injury, i.e., a pressure ulcer, skin tear, or blister?

### Plantar skin deforms less under load than nonplantar skin

Pressure ulcers form in soft tissue exposed to excessive deformations as a result of several overlapping pathways, including ischemia, reperfusion, poor lymphatic drainage, and direct cell deformation (*1*). Plantar skin is exposed to some of the highest mechanical loads on the body. Despite this exposure to mechanical loads, pressure ulcers rarely form in plantar skin. To explain plantar skin’s resistance to pressure ulcers, we hypothesized that plantar skin would deform less under equivalent loads than nonplantar skin. To test this hypothesis, we quantified the deformation of ex vivo human skin under load.

**Fig. 3:**
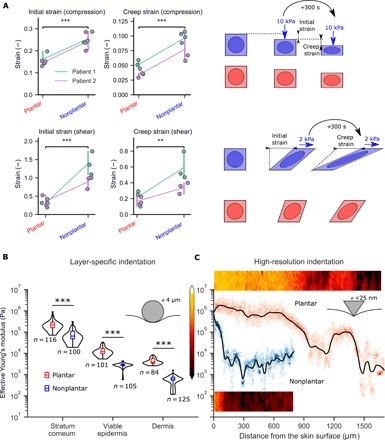
Plantar skin resists deformation. (**A**) Uniaxial compression and simple shear tests on ex vivo skin. Deformation was measured as the initial strain after compression of 10 kPa (top) and shear of 2 kPa (bottom). Tests are from two patients, with three samples from each anatomical location. Loads were maintained for 300 s, and final deformation was measured as creep strain. (**B**) AFM indentation experiments using a spherical (4 μm) tip on cryostat sections of skin. (**C**) High-resolution force mapping using a sharp AFM tip shows that the change in Young’s modulus with depth is more gradual in plantar skin. Two-dimensional (2D) stiffness maps (50 μm wide) are shown alongside depth-specific data. Black lines represent LOESS regression fits of the data, while color intensity represents the spread of elastic moduli at each depth. ***P < 0.001, **P < 0.01, two-sided Student’s *t* test.

We obtained plantar and nonplantar human skin samples from two patients and extracted three 8-mm-diameter specimens from each skin sample. Each specimen was exposed to a uniaxial compressive load of 10 kPa and a simple shear load of 2 kPa (fig. S2) using a micromechanical testing device. These stresses were selected as they are at the upper range of nonplantar skin tolerance. The micromechanical testing device measured applied loads and displacements from which tissue deformations were quantified by measuring the strain immediately after the target load was reached (referred to as initial strain). These tests revealed that plantar skin deforms 1.6-fold less (*P* < 0.001) than nonplantar skin under compression and 3.4-fold less (*P* < 0.001) under shear (Fig. 3A), in support of our hypothesis.

Soft tissues continue to deform under sustained loads due to viscoelastic and poroelastic effects (*30*) in a phenomenon known as “creep.” Sustained loading is a common cause of pressure ulcers (*1*), and so, quantifying the creep response of skin is crucial to understanding its load tolerance. To quantify the creep response of both plantar and nonplantar skin, we subjected the same samples to a sustained load for 300 s and measured long-term deformation (referred to as creep strain). Under sustained load, plantar skin underwent 2-fold less creep strain (*P* < 0.001) compared to nonplantar skin in compression and 2.4-fold less in shear (*P* < 0.01; Fig. 3A). The above analyses demonstrate the ability of plantar skin to resist deformation at the whole-skin level and over extended loading periods.

We next sought to quantify how the distinct composition of plantar epidermis and dermis affects each layer’s deformation resistance. To do this, we quantified the “effective Young’s modulus” (a measure of a structure’s resistance to deformation) of each individual layer using an atomic force microscope (AFM). We obtained 100-μm-thick sections of plantar and nonplantar skin from two patients. A spherical AFM probe (tip diameter, 4 μm) was used as a nanoindenter to generate highly localized force-displacement curves (fig. S2). The force-displacement curves were analyzed using Hertzian contact theory to extract an effective Young’s modulus at each test location. We obtained effective Young’s modulus measurements at locations within the stratum corneum, the viable epidermis, and the dermis. We found that each of the stratum corneum, viable epidermis, and dermis layers had a significantly larger Young’s moduli in plantar skin than in nonplantar skin (by a factor of 3, 4.8, and 7.2, respectively; Fig. 3B). These results suggest that all layers of plantar skin contribute to its overall deformation resistance. They also show that there is a substantial mismatch in Young’s modulus between skin layers.

Interfaces between materials with mismatched Young’s moduli are known to create stress concentrations—local regions that experience stresses far higher than the global mean stress (*31*). To analyze the interfaces between the epidermis and dermis in greater detail, we used a pyramidal AFM probe (tip diameter, <25 nm). The smaller contact area of this probe enabled us to create high-resolution stiffness maps through the thickness of the skin (Fig. 3C). We found that the change in Young’s modulus with depth is more gradual in plantar skin than in nonplantar skin (Young’s modulus drops 18% per 100 μm in plantar skin compared to 84% per 100 μm in nonplantar skin). This more gradual change in Young’s modulus with depth may help to mitigate the stress concentration effect in plantar skin, eliminating “hotspot” areas of stress where ulceration or tearing could be initiated under loading conditions.

In summary, plantar skin has a measurably enhanced resistance to deformation in both compression and shear and over extended loading periods. This resistance to deformation could plausibly protect plantar skin from pressure ulcers. Furthermore, each layer of plantar skin exhibits enhanced resistance to deformation compared to nonplantar skin.

### Plantar skin is protected from high local deformations under load

When skin is externally loaded, deformations are heterogeneously distributed within it due to its complex morphology and heterogeneous composition (*15*). This heterogeneity can lead to localized regions of pathologically high deformations, even if the whole-skin deformations are low. We therefore wanted to quantify local deformations in plantar skin in greater detail than was possible using our mechanical tests. To do this, we developed finite element models of plantar and nonplantar skin under load. Finite element analysis provides a computational technique to calculate the distributions of deformations and forces within a load-bearing structure. The key steps in finite element analysis are to (i) define the geometry and material properties of the structure, (ii) assign loads and boundary conditions, and (iii) solve the resulting differential equations that govern the distribution of deformations within the structure.

We defined the geometry of plantar and nonplantar skin using the same images of H&E-stained sections that were previously used to quantify skin morphology (Fig. 2A). These images were segmented into the stratum corneum, the viable epidermis, and the dermis. The resulting geometry represents a 4-mm-wide transverse section of skin (fig. S3C), referred to as the region of interest (ROI).

We defined the composition (material properties) of the stratum corneum, viable epidermis, and dermis using both the whole-skin mechanical testing data and the layer-specific effective Young’s moduli obtained using AFM. We use the term “composition” to refer to these material properties throughout the rest of the paper to maintain consistency. Briefly, Ogden hyperelastic models were fitted to the plantar and nonplantar whole-skin data. To infer each layer’s material properties, we scaled this whole-skin model based on both the thickness of each layer and its relative stiffness. Relative stiffness was measured by comparing the effective Young’s moduli of each layer (from our AFM data), while thickness was measured from histological images, as in Fig. 2A. Together, this approach balances the need to represent the relative stiffness of each layer as well as the response of the whole skin while overcoming the need to dissect individual layers for testing.

To model the loads and boundary conditions experienced by skin in vivo, we attached the ROI to simplified representations of the surrounding skin and soft tissue (fig. S3). We then applied compressive and shear loads of 10 kPa to a flat surface in contact with the skin. Using this approach, we could calculate the distribution of deformations throughout the skin by calculating the (maximum) shear strain at each location. Shear strain is commonly used to quantify deformations in soft tissue, and regions of tissue under high shear strain have been shown to correlate with injuries such as pressure ulcers in vivo (*11*, *12*).

**Fig. 4:**
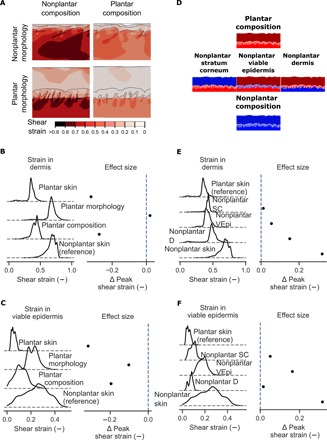
Plantar skin experiences less deformation than nonplantar skin under load. (**A**) Shear strains in nonplantar skin (top left) and plantar skin (bottom right) under equal compressive and shear load, with models representing plantar morphology, but nonplantar composition and vice versa. Contour plots show the highest shear strains induced in the dermis of nonplantar skin. (**B**) Kernel density estimates of shear strains in the dermis and (**C**) viable epidermis with effect size relative to nonplantar skin. (**D**) Three knockout models were created from the original plantar model in which the material properties of the stratum corneum (left), viable epidermis (middle), or dermis (right) were reduced from plantar to nonplantar. (**E**) Kernel density estimates of shear strain in the dermis and (**F**) viable epidermis for each of the knockout models. Peak shear strains are statistically significantly different between all models in this analysis (*P* < 0.0001, two-sided Student’s *t* test) due to the very high number of sampling points (*n* = 5402 and 8845 for plantar and nonplantar models, respectively). Because the number of sampling points for these simulations is arbitrary, effect size is reported in this figure rather than statistical significance.

We used these finite element models to compare the microstructural deformations within plantar and nonplantar skin under equivalent compressive and shear loads (Fig. 4A). We found that the peak shear strains in both the viable epidermis and dermis were lower in plantar skin compared to nonplantar skin (4.6- and 1.7-fold lower in viable epidermis and dermis, respectively, compared to nonplantar skin; Fig. 4, B and C), showing that plantar skin’s deformation resistance at the whole-skin level also translates to the microstructural level.

The spatial distribution of shear strains within the dermis was also different between plantar and nonplantar skin. In nonplantar skin, large regions of high shear strain penetrate deep into the dermis, while regions of high shear strain were isolated to the tips of rete ridges in plantar skin (Fig. 4A). Large regions of high shear strain could potentially restrict microcirculation to the skin and increase the risk of ischemic damage. In summary, computational models of skin under load predict that plantar skin is protected from extreme local deformations, complementing the whole-skin deformation resistance found experimentally.

### Plantar skin composition enhances its resistance to deformation-induced injury

Our histological analyses show that plantar skin has distinct morphology and composition, while our mechanical tests show that plantar skin has enhanced resistance to deformation. However, these analyses cannot determine whether morphology or composition contributes most to plantar skin’s enhanced load tolerance. The computational models introduced in the previous section enable us to integrate morphological and compositional data, as well as selectively alter the model in silico. Using this approach, we sought to quantify the relative effects of plantar skin morphology and composition on its load tolerance.

To quantify the role of morphology in reducing deformation, we modeled skin with a plantar morphology, but nonplantar composition (material properties), and compared this to the model of nonplantar skin as a reference. Comparing the dermis of both models, we found similarly high peak shear strains in skin with plantar morphology and nonplantar composition compared to nonplantar skin (0.81 versus 0.77, respectively; Fig. 4B)—both substantially higher than plantar skin (0.49). Comparing the viable epidermis of both models, peak shear strains were lower in skin with a plantar morphology compared to nonplantar skin (0.3 versus 0.4, respectively), but not to the same extent as plantar skin (0.08; Fig. 4C). These results indicate that skin with a plantar morphology, but nonplantar composition, experiences deformations that are more like nonplantar skin than plantar skin, demonstrating that plantar morphology does little to protect skin from deformation.

To quantify the role of composition in reducing deformation, we modeled skin with plantar composition and nonplantar morphology and again compared it to the nonplantar skin model as a reference. Comparing the dermis of both models, we found that shear strains in skin with plantar composition and nonplantar morphology were lower compared to nonplantar skin (0.53 versus 0.77, respectively; Fig. 4B) and similar to plantar skin (0.49). This trend was also found in the viable epidermis, where skin with a plantar composition and nonplantar morphology experienced peak deformations of 0.2, which was more similar to plantar skin than nonplantar skin (0.08 in plantar skin and 0.4 in nonplantar skin). This result indicates that the composition of plantar skin accounts for a large proportion (85% in the dermis and 63% in the epidermis) of the deformation protection observed in plantar skin.

The above analyses indicate that plantar skin’s composition contributes more than its morphology to its deformation resistance. This deformation resistance could originate in the distinct collagen structure of the dermis, the unique cytoskeletal structure of the epidermis, or both. Our microscale mechanical tests in the previous section also showed that the Young’s moduli are different across the stratum corneum, viable epidermis and dermis. To clarify the role of each specific layer’s composition in reducing deformations, we developed “knockout” variants of the plantar skin model in which the material properties of one of the three skin layers were reduced to that of nonplantar skin (Fig. 4, D to F). The greatest increase in strains in the viable epidermis occurred with a “knocked-out” viable epidermis (2.9-fold; Fig. 4F), followed by a knocked-out stratum corneum (1.6-fold). A knocked-out dermis had a comparatively small effect on strains in the viable epidermis (1.2-fold). In the dermis (Fig. 4E), the greatest increase in strains occurred with a knocked-out dermis (1.4-fold), with the knocking out of viable epidermis and stratum corneum having relatively lower effects (1.1- and 1.03-fold, respectively).

Collectively, these results show that the dermis and viable epidermis of plantar skin are protected from excessive deformations by their respective compositions and that the morphology of plantar skin alone is insufficient to protect from deformation-induced injury such as pressure ulcers.

### A thick stratum corneum provides morphological protection from stress-induced injury

In the previous section, we quantified the distribution of deformations within skin using finite element models, furthering our understanding of deformation-induced injuries such as pressure ulcers. Another mode of injury occurs when the stresses in skin cause ruptures, which can manifest as a skin tear in the dermis (*6*) or a blister or abrasion in the viable epidermis (*32*). The von Mises stress criterion is a common method to quantify the stresses that lead to rupture in polymers (*33*) as well as biological soft tissues (*34*). Von Mises stress can be calculated from finite element models such as those described in the previous sections, providing us with information about the plantar skin’s ability to resist stress-induced injury.

**Fig. 5:**
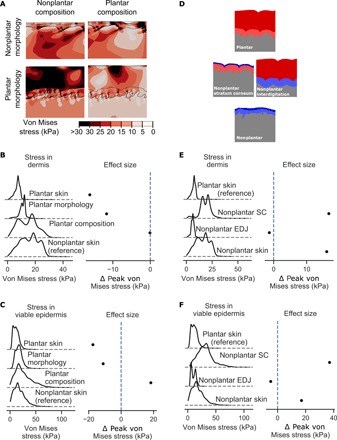
Plantar skin morphology protects it from stress-induced injury. (**A**) Stresses induced in plantar (bottom right) and nonplantar skin (top left) under equal compressive and shear loads. Contour plots show the distribution of von Mises stresses in the skin. Plantar composition (via the material properties) was combined with nonplantar morphology (top right) and vice versa (bottom left). (**B**) Kernel density estimates showing the distribution of stress magnitudes in the dermis and (**C**) viable epidermis. Difference in peak stress (defined as 95th percentile value) between each model and nonplantar skin is also shown (lines indicate 95% bootstrapped confidence interval of the difference). (**D**) Knockout models of plantar skin were created by reducing the thickness of the stratum corneum to that of nonplantar skin or reducing the interdigitation between the epidermis and dermis. (**E**) Kernel density estimates of the stress in the dermis and (**F**) viable epidermis for knockout models. Difference in peak stress is presented relative to plantar skin. Peak von Mises stresses are statistically significantly different between all models in this analysis (*P* < 0.0001, two-sided Student’s *t* test) due to the very high number of sampling points (*n* = 5402 and 8845 for plantar and nonplantar models, respectively). Because the number of sampling points for these simulations is arbitrary, effect size is reported in this figure rather than statistical significance.

To evaluate whether plantar skin is resistant to tears, we first quantified the von Mises stresses induced in plantar and nonplantar skin using the same finite element models as described in the previous section (Fig. 5A). Using these models, we found that peak von Mises stresses were lower in both the dermis and viable epidermis of plantar skin relative to nonplantar skin under equal compressive and shear loads (2.7- and 1.8-fold lower in the dermis and viable epidermis, respectively; Fig. 5, B and C).

To quantify the role of morphology in reducing von Mises stress, we modeled skin with a plantar morphology and nonplantar composition and compared to the nonplantar skin model as a reference (Fig. 5, B and C). These models revealed that skin with a plantar morphology and nonplantar composition experienced lower peak von Mises stress relative to nonplantar skin (1.8- and 1.4-fold reduction in the dermis and viable epidermis, respectively), albeit not to the same extent as plantar skin. This indicates that the morphology of plantar skin partially contributes to its protection against stress-induced injury.

To quantify the role of composition in reducing von Mises stress, we modeled skin with plantar skin composition and nonplantar morphology and compared it to nonplantar skin as a reference. In skin with plantar skin composition and nonplantar morphology, peak von Mises stresses in the dermis reached a similar level to nonplantar skin (25.6 kPa in both models). The viable epidermis of skin with plantar skin composition alone experienced substantially higher peak von Mises stress than even nonplantar skin (57.9 versus 37.6 kPa). These results indicate that plantar composition alone cannot reduced the stresses experienced within skin.

The above analyses indicate that plantar skin’s morphology contributes more than its composition to protect against stress-induced injuries, such as a skin tear. Because plantar skin morphology has two distinct characteristics—a thick stratum corneum and greater interdigitation between the epidermis and dermis—we wanted to determine which of these morphological features is most protective against stress-induced injury. For this, we developed knockout models of plantar skin in which either the thickness of the stratum corneum or the level of interdigitation was reduced to that found in nonplantar skin (Fig. 5D). Knocking out interdigitation had the effect of decreasing the stresses in the dermis (from 9.6 to 8.3 kPa; Fig. 5E) and viable epidermis (from 21.7 to 16.9 kPa; Fig. 5F), indicating that interdigitation does not protect from stress-induced injury. In contrast, knocking out the stratum corneum, creating an interdigitated skin with a thin stratum corneum, created stresses substantially higher than those observed even in nonplantar skin (from 9.6 to 26.6 kPa in the dermis and from 21.7 to 58.4 kPa in the viable epidermis), indicating a particularly injurious morphology.

Collectively, these results show that plantar skin is better protected from von Mises stress than nonplantar skin, with the thick stratum corneum of plantar skin providing the most protection. The presence of this low-stress environment helps to explain the reduced risk of stress-induced injuries such as tears in plantar skin.

## DISCUSSION

Skin is prone to injury when exposed to external mechanical loads, yet skin differs greatly in the amount of load it can tolerate depending on its anatomical location. This anatomical variability in load tolerance offers the potential to uncover the properties of skin that determine load tolerance. However, it has been difficult to quantify the roles of specific skin properties because many of them exist together in load-tolerant skin. Here, we overcame this problem by combining multiscale mechanical characterization with computational models of load bearing. We found that both the morphology and composition of plantar skin enhance its load tolerance in different ways. More specifically, the thick stratum corneum of plantar skin protects it against stress-induced injuries such as skin tears and blisters, whereas plantar skin’s epidermal and dermal compositions protect against deformation-induced injuries such as superficial pressure ulcers.

The thick stratum corneum of plantar skin is commonly thought to enable locomotion without injury (*35*). Our results corroborate this hypothesis, showing that a thick stratum corneum reduces stresses in the underlying tissue. The stratum corneum of plantar skin thickens to form a callus in response to loads (*36*), and the stress reductions quantified here support the evolutionary benefits of broadly distributed calluses (*35*).

The interdigitated EDJ has also been suggested to provide protection from injury, particularly shear loads (*37*). Our results do not support this role for interdigitation, because both stresses and strains within both the epidermis and dermis were higher in skin with interdigitation in the absence of a thick stratum corneum. The greater interdigitation found in plantar skin may instead support greater nutritional demands of the thick plantar epidermis (*38*) or tactile sensation (*39*). We found that the worst-case scenario was that of skin with a thin stratum corneum and greater interdigitation—a scenario similar to psoriatic skin. This finding suggests that amplified stresses and strains could play a role in the etiology of psoriasis and merits further study.

Both morphological and compositional changes have been implicated in the increase in skin fragility with age (*40*). Our findings suggest that these changes may influence distinct injury types. The epidermis thins with age, as the proliferative activity of basal keratinocytes reduces. Our results suggest that this thinning of the epidermis, and thus the change in morphology, would create vulnerability to skin tears and blisters but not pressure ulcers. Instead, the composition of aged skin, such as its decreased collagen and elastin synthesis, would alter the material properties of its dermis, increasing vulnerability to pressure ulcer formation.

A common target for skin regeneration strategies such as autografting or tissue-engineered skin substitutes is restoring a more natural skin morphology (*41*). Our findings show that while efforts to improve the morphology of the regenerated skin is important, the composition of the regenerated skin layers should also be targeted. Regenerative therapies that can induce a robust collagen matrix structure or an enhanced cytoskeletal network may help to restore load-bearing function after plantar injuries such as diabetic ulcers and could even augment skin that is required to bear load, such as on the residual limb of an amputee.

We chose to study the plantar region because it is a particularly load-tolerant anatomical location. However, injuries to the surrounding region do occur. For example, the back of the heel is one of the most common locations of pressure ulcers, particularly in supine patients (*1*). The vulnerability of the human heel can be explained by pointing out that the border between plantar and nonplantar skin is low down on the human heel, leading to nonplantar skin bearing the high contact pressures in supine patients. The border between the stiff plantar skin and less-stiff nonplantar skin may exacerbate heel ulcers.

This study highlights the importance of quantifying mechanics at the microstructural level. Our microstructural models show that the deformations and stresses within the skin are highly heterogeneous and that external loads can be either amplified or attenuated, depending on the skin microstructure. These findings agree with recent experimental work, showing higher shear strains in dermal tissue relative to the epidermis (*42*). We have captured this heterogeneity by explicitly modeling the morphology of skin. Other researchers have instead focused on the whole-skin (*43*) response to load, potentially allowing much larger anatomical regions to be modeled at the expense of microstructure. In the future, computational models of skin should be extended to real-world loading scenarios using multiscale models to account for microstructure.

There are some limitations to this study. Our study focuses on the epidermal and dermal layers of skin and is therefore more relevant for superficial pressure ulcers than for deep tissue injuries. We have also focused here on showing that plantar skin experiences lower stresses and strains under load than nonplantar skin. As well as mechanisms to reduce stresses and strains, plantar skin may also have mechanisms to tolerate high stresses and strains that warrant further study. For example, the mechanical strength of its constituents could be different, as could the rate at which damage accumulates within, or is removed from, the tissue. We have also only analyzed one nonplantar region—the lower limb—while load tolerance is likely to vary across nonplantar regions. More detailed load tolerance information across anatomical locations is needed.

The experiments performed in this study are not exhaustive, and more research is needed to fully characterize both plantar and nonplantar skin’s load response. We chose to analyze the whole skin under simple load cases and in an ex vivo setup, enabling us to quantify differences between plantar and nonplantar skin. Future work could extend these analyses to more complex load cases.

The computational models used in this study were based on ex vivo experiments at the whole-skin level and within each layer. This approach enabled us to integrate microstructural material properties with overall skin response and was suitable for our objective of comparing two anatomical locations. With further improvements in these techniques, there is the potential to more accurately predict skin injury. Some improvements could include incorporating collagen structure using anisotropic material models, moving to a three-dimensional (3D) representation of skin structure, and incorporating time-variant material properties.

In conclusion, this body of work quantifies the contributions of morphology and composition to plantar skin’s load tolerance, indicating that each property plays a distinct but complementary role. The work indicates that a thick stratum corneum is most important to protect skin from stress-induced injuries such as skin tears and blisters, while the composition of each skin layer is most important for protection against deformation-induced injuries such as pressure ulcers. The combined approach of multiscale mechanical testing and computational modeling can now be extended to investigate age-related skin changes and to enhance the load tolerance of engineered skin substitutes.

## MATERIALS AND METHODS

### Histology and morphometrics

Plantar and nonplantar skin was excised from an amputated lower limb within 3 hours of surgery. National Health Service Research Ethics Committee–approved consent forms (approval 17/W/0161) were used to obtain ethical approval, and the tissue was routed through the Imperial College Healthcare Tissue Bank (Human Tissue Authority license 12275). Excised skin was embedded in OCT (optimal cutting temperature) embedding medium and stored at −80°C until needed. For morphometric imaging, 20-μm-thick sections of both plantar and nonplantar skin were cut on a cryostat and stained using H&E. These sections were imaged on a Zeiss inverted microscope at ×5 magnification. Images were semiautomatically stitched using Fiji plugin MosaicJ to create full-section images covering an area of approximately 7 mm × 3 mm. These images were semiautomatically segmented using the “versatile wand” tool in Fiji (*44*) to define the boundaries of the stratum corneum and viable epidermis. The resulting binary images were imported to Inkscape (v0.91), where the region outlines were extracted as vectorized line segments. The resulting file was imported into AutoCAD, where coordinates of the curves defining the layer boundaries were converted to csv files. These were imported into a Jupyter computational notebook where the package geopandas was used to quantify the thickness of each layer and the tortuosity (see notebook in the Supplementary Materials). This allowed the perpendicular distance between two curves to be measured at regular intervals along the skin surface. Tortuosity was measured as the arc-chord ratio. This was calculated by dividing the curve defining the EDJ into equal length sections and dividing the arc length along the EDJ by the end-to-end distance for each section.

The same tissue samples were sectioned at 100 μm for SHG imaging of plantar and nonplantar collagen structure. All images were preprocessed using a prerecorded Fiji Macro, including setting minimum-maximum at (0, 4000), applying the Gaussian blur filter (σ = 0.50), and subtracting background (rolling ball radius, 40 pixels). Four ROIs of 600 μm × 450 μm were then selected from tile scans of approximately 3000 μm × 2000 μm. Stacks were then separated into single images. An interactive learning and segmentation open source software called ilastik was applied to facilitate image segmentation based on the pixel classification mode (*45*).

To describe the distribution of collagen fibers in the dermis, fibers were divided into two types: thin fibers (≤10 μm) and thick fibers (>10 μm). As part of the ilastik workflow, training was performed on a set of six images to define pixels as belonging to one of the three features: thin fibers, thick fibers, and background (fig. S1). A mask for the segmentation of collagen fibers was subsequently applied to 20 images (single *Z*-stack steps) in a batch processing mode. The segmented images were exported as .tif files to be used in further analysis in Fiji. We used the Fiji Macro Recorder to automatize the separation of segmented channels, quantification of the pixel area covered by each of the three features (thin fibers, thick fibers, and background), and measurement of the fiber thickness with BoneJ plugin for ImageJ (*46*). On the basis of the pixel area covered by each of the three features, the total proportion of collagen in the dermis and the proportion of thin/thick collagen fibers were calculated.

The orientation of collagen fibers was assessed using an open source MATLAB software CurveAlign (*47*). We used the same ROIs of 600 μm × 450 μm selected from tile scans as used for the assessment of proportion and thickness of collagen fibers. The primary settings with fraction of coefficient to keep = 0.06 and distance from boundary to evaluate = 150 pixels were used.

The same tissue samples were sectioned for immunofluorescence and imaged on widefield inverted microscope (Zeiss Axio Observer). Sections were taken at 10 μm for collagen IV + loricrin and K9 + laminin-α3/laminin-5 and at 6 μm for DSG1 + collagen 1 antibodies. Sections were fixed using 4% paraformaldehyde (AGR1026, Agar Scientific) in phosphate-buffered saline (PBS) at room temperature (RT) for 10 min. PBS containing 5% goat serum (S-1000, Vector Laboratories) and 0.3% Triton X-100 (X-100, Sigma-Aldrich) was used for blocking and permeabilization of tissue sections at RT for 30 min. Primary antibodies diluted in 5% goat serum and 0.3% Triton X-100 in PBS (table S1) were then added to the sections and incubated overnight at 4°C after washing with PBS. The sections were then washed three times in 0.1% Triton X-100 diluted in PBS and were incubated in secondary antibodies diluted in PBS for 1 hour at RT in the dark. Secondary antibodies included Alexa Fluor 488 goat anti-mouse, 594 anti-rabbit, 546 anti-mouse, and 488 anti–guinea pig (A11001, A11012, A11030, and A11073, respectively; 1:500; Thermo Fisher Scientific). After three washes in PBS, the tissue sections were mounted on the glass slides using mounting medium containing DAPI (4′,6-diamidino-2-phenylindole) (H-1200, Vector Laboratories) as a nuclear stain.

### Mechanical testing

Skin was acquired from cadaveric lower extremities of two patients obtained from a licensed human tissue facility. Ethical approval for this study was obtained from the Tissue Management Committee of the Imperial College Healthcare Tissue Bank (ethical approval number: 12/WA/0196). The cadaveric specimens were provided by a licensed human tissue facility, and the tissue donors had consented to their use for scientific research. Tissue was stored at −20°C until testing. An 8-mm-diameter biopsy punch was used to excise cylindrical samples from two sites: the ventral foot and beneath the first metatarsal. Three samples from each site were excised. The samples were trimmed of subcutaneous tissues, and the cylindrical sample was placed between the platens of a multiaxis mechanical testing device (Biomomentum Mach-1 v500 cst). A six-axis force transducer (ATI nano 17 F/T18874, 12.5-mN force resolution) was used to measure normal and shear forces. The surfaces of the platens were lined with sandpaper (400 grade) to enable enough grip for shear testing. To maintain the hydration of the sample, gauze was placed around the test specimen (fig. S2A), and PBS solution was applied to the gauze.

To control the strain rate of experiments, the moveable platen was brought into contact with the sample, allowing the height of the sample to be measured. Velocity in millimeters per second was then calculated to ensure a constant strain rate $\left(\frac{\Delta l}{l}/s\right)$ based on the sample height (*l*). Nominal stress was calculated as the force divided by the sample cross-sectional area (16π mm^2^).

The samples were loaded in compression at a strain rate of 0.005 s^−1^ to a maximum stress of 10 kPa. Three preconditioning cycles were performed, followed by a fourth from which the data were analyzed. After the fourth load cycle, the force was held constant for a period of 300 s to assess creep deformation. The samples were then loaded in simple shear (displaced in the plane of the sample). Three preconditioning cycles were performed at a strain rate of 0.005 s^−1^ to a shear stress of 2 kPa. The fourth cycle was recorded, and the stress was maintained for a period of 300 s as before. The strains at the beginning and end of the hold cycle (representing initial and creep strain, respectively) were recorded.

Tests were performed on three samples from each body site and repeated for two patients. Data analysis was performed in a Jupyter computational notebook. Each test was split into its ramp and hold sections, and the strains at the beginning and end of the hold section were recorded. Elastic strain was calculated as the strain at the beginning of the ramp section, while creep strain was calculated as the change in strain over the hold section. Student’s *t* test was used to test for differences between plantar and nonplantar data (patient data were pooled, giving *n* = 6 for each comparison).

### AFM measurements

From the tissue samples described in the “Histology and morphometrics” section, 30-μm-thick sections of both nonplantar and plantar skin were analyzed using an AFM (JPK NanoWizard 4). The sections were tested under aqueous conditions in PBS solution. Two modes of testing were used: quantitative imaging, which allows high-speed testing and mapping across an ROI, and force spectroscopy, which allows greater control over the indentation displacement profile.

Quantitative imaging tests (*48*) were performed on regions of 50 μm width and 100 μm depth. The ROI was then moved progressively away from the skin outer surface to provide a mapped region of 50 μm × 1800 μm and 50 μm × 800 μm for plantar and nonplantar samples, respectively. A rectangular cantilever (ContGB-G; budgetsensors.com) with nominal spring constant of 0.2 N/m and pyramidal tip was used. Actual spring constant was measured using the thermal method in air (*48*). Sensitivity was measured by indenting glass under aqueous conditions. Each 50 μm × 100 μm region consisted of 32 × 64 individual indentations, from which the force-displacement data were calculated. The force-displacement curves were analyzed using JPK data processing suite (version 6.1.22). After subtraction of the baseline force and adjustment to calculate vertical tip displacement and contact point, a Hertzian contact model was fitted to the extend portion of the force-displacement curve, which enabled a structural or effective Young’s modulus (*E*) to be calculated at each point. The data were then stitched and processed using MATLAB to create 2D stiffness maps. These maps were then processed in a Jupyter computational notebook to determine how the effective modulus varies with depth (see the Supplementary Materials).

Force spectroscopy was used to analyze the mechanical response more precisely than can be achieved with quantitative imaging and a sharp tip. Instead, a spherical tip with a diameter of 4 μm and a spring constant of 0.2 N/m was used. A ramp of 1.5 μm/s to force set point of 30 nN, followed by a retract phase, was defined. The same protocol as above was used to calculate the effective Young’s modulus for each curve. Between 70 and 120, these tests were taken at each layer (stratum corneum, viable epidermis, and dermis, identified with an optical microscope) and in both plantar and nonplantar samples. Tests were repeated on samples from two patients. Results from patient 1 are reported in the main text, and those from patient 2 are reported in fig. S2.

### Microstructural model

Finite element models of plantar and nonplantar skin were developed from the H&E histological images, with each step detailed below.

#### Geometry

The curves defining the boundaries between the stratum corneum, viable epidermis, and dermis (described in the “Histology and morphometrics” section) were imported to Abaqus finite element software. They were used to define the 2D geometries of the three skin layers for both plantar and nonplantar skin. The geometry was meshed with quadrilateral, hybrid finite elements to produce a minimum mesh characteristic length of approximately 15 μm (fig. S3D).

#### Material properties

Material properties for the three skin layers were derived from both macro- and microscale testing of nonplantar and plantar skin. First, an Ogden hyperelastic model was fitted to the uniaxial compression and simple shear data (see the “Mechanical testing” section) using the MCalibration curve fitting software (v5.0.1, Veryst Engineering; fig. S3A). This analysis provides the whole-skin mechanical response but not individual skin layers. To derive the properties of each individual layer, we used a Reuss or “rule-of-mixtures” model (*49*). The rule-of-mixtures approach was initially developed to study the properties of composite structures with two or more constituents. The overall response of such a composite loaded perpendicular to the layers of the material is assumed to depend on (i) the volume fraction of each constituent and (ii) the relative Young’s modulus of each constituent. Typically, the overall response of the composite is calculated from the known properties of each constituent. In the case of skin, we can say that the overall tissue-level shear modulus, μ, depends on the behavior of individual layers (μ_d_, μ_v_, and μ_sc_ for the dermis, viable epidermis, and stratum corneum, respectively)

$$\mu ={\left[\frac{f_{s}}{\mu_{s}}+\frac{f_{v}}{\mu_{v}}+\frac{1-f_{s}-f_{v}}{\mu_{d}}\right]}^{-1}$$

where *f*_s_ and *f*_v_ are the volume fractions of stratum corneum and viable epidermis, respectively. However, in our case, we know the overall response and want to infer the response of each layer. To do this, we estimated the volume fractions of each layer as its mean thickness divided by the full-skin thickness. After this step, there are three unknowns remaining (the stiffnesses of each layer μ_d_, μ_v_, and μ_sc_). We can reduce this to one unknown using the effective Young’s moduli found in AFM. While these microscopic measurements cannot be directly inputted into a whole-tissue model, we made the assumption that the relative stiffnesses at this microstructural level translate to the whole-skin level, i.e., that the effective Young’s moduli *E*_SC_:*E*_V_:*E*_D_ measured in AFM are in the same ratio as the shear moduli μ_s_:μ_v_:μ_d_ in the finite element models. Under these assumptions, the stratum corneum and viable epidermis shear moduli can be related to the dermis shear modulus (μ_s_ = *s*μ_d_ and μ_v_ = *v*μ_d_, where *s* and *v* are the scale factors $\frac{E_{\text{SC}}}{E_{D}}$ and $\frac{E_{V}}{E_{D}}$, respectively), the last remaining unknown. The above equation, when rearranged for μ_d_

$$\mu_{d}=\mu [\frac{f_{s}}{s}+\frac{f_{v}}{v}+1-f_{s}-f_{v}]$$

The final calculated shear modulus values for each layer and within each skin type are given in table S2. Exponents for the Ogden model were −13.04 and −14.53 for nonplantar and plantar skin, respectively, based on the curve fitting from the MCalibration software.

#### Boundary conditions

When skin is excised using a biopsy punch, the sample tends to shrink and curl because of the release of in vivo tension from the surrounding tissue (fig. S3). To simulate the in vivo loading environment, we developed a simulation pipeline to restore the in vivo loaded configuration. First, the skin geometry, defined as described above, was attached to peripheral skin of an idealized geometry to ensure that the boundary of the model was sufficiently far from the analyzed area. A prestretch step was then performed in which the curvature of the sample was reduced, straightening the histologically derived region to induce a prestretch of 1.2. To simulate the reaction forces of the underlying soft tissue, a region with material properties representing subcutaneous tissue (Ogden hyperelastic material, μ = 25 kPa, α = 5) (*16*) was defined beneath the skin, and tied contact was assumed between this subdermal layer and the dermis.

The skin was then loaded using a rigid surface, which was displaced into contact with the skin, and then displaced transversely. Rough contact was assumed between the skin and the interacting surface, preventing relative motion at the surface and inducing shear deformations in the skin. Both skin models were loaded to the same maximum compressive and tangential load (0.1 N/mm in both directions equivalent to approximately 10 kPa of normal pressure and shear). Models were constructed using Abaqus/CAE and solved using Abaqus/standard.

#### Outputs

The shear strains and von Mises stresses were calculated at every integration point (four per element). The region of the dermis to a depth of 500 μm was defined as an ROI and analyzed across all models. The field variables (stress and strain tensors and surface tractions) were extracted at the end point of the simulation, when both compressive and shear loads are applied. These field variables were calculated relative to the in vivo unloaded configuration, i.e., the stresses, strains, and tractions induced by prestretching the model are not added to the stresses induced by external loading. Using the in vivo unloaded configuration as our reference state, we ensured that we are considering the effect of external loading only. There is evidence to support this approach. For example, the desmosomes that connect keratinocytes have been shown to bear tension when external load is applied, but not in the case of no external load (*29*).

Because the density of elements in our finite element mesh is heterogeneous, calculating summary statistics on the raw field variables would produce values weighted toward small elements. To overcome this, we used the Python module Pandas to resample the field data by weighting each integration point by its associated volume. This ensured that stress and strain distributions were independent of mesh density and heterogeneity. Kernel density estimates of the data were computed using StatsModels (www.statsmodels.org), and effects sizes were computed by bootstrapping the difference in the peak value (*50*). The peak value was defined as the 95th percentile of the data to avoid outlier values that may occur because of boundary conditions or mesh transition.

#### Model variants

Four models were initially analyzed: plantar morphology with either plantar or nonplantar material properties and nonplantar morphology with either plantar or nonplantar material properties. The plantar skin model was then adapted to create knockout models (fig. S3C). One such model involved reducing the thickness of the stratum corneum to approximately that encountered in nonplantar skin. Another involved reducing the level of interdigitation between the epidermis and dermis. Further models were created, where one of the layers’ properties was reduced to those of nonplantar skin. All geometries created are included as Abaqus input files in the Supplementary Materials.

## Supplementary Material

http://advances.sciencemag.org/cgi/content/full/5/10/eaay0244/DC1

Download PDF

Morphology and composition play distinct and complementary roles in the tolerance of plantar skin to mechanical load

## SUPPLEMENTARY MATERIALS

Supplementary material for this article is available at http://advances.sciencemag.org/cgi/content/full/5/10/eaay0244/DC1 Table S1. Antibodies used. Table S2. Shear moduli based on rule-of-mixtures analysis. Fig. S1. Histological analysis. Fig. S2. Mechanical testing of skin. Fig. S3. Constructing finite element models of the skin.
