# Supplementary material for: Morphology and composition play distinct and complementary roles in the tolerance of plantar skin to mechanical load
Source: Sci Adv. 2019 Oct 9;5(10):eaay0244. doi: 10.1126/sciadv.aay0244 (PMC6785259; doi:10.1126/sciadv.aay0244)
Supplement: http://advances.sciencemag.org/cgi/content/full/5/10/eaay0244/DC1 [file supp_5_10_eaay0244__index.html]

Science Advances | Science AdvancesAAASSearchScience AdvancesMenu

## Supplementary Materials

**This PDF file includes:**

- Table S1. Antibodies used.
- Table S2. Shear moduli based on rule-of-mixtures analysis.
- Fig. S1. Histological analysis.
- Fig. S2. Mechanical testing of skin.
- Fig. S3. Constructing finite element models of the skin.

Download PDF

**Files in this Data Supplement:**

- Adobe PDF - aay0244\_SM.pdf
