# Supplementary material for: Morphology and composition play distinct and complementary roles in the tolerance of plantar skin to mechanical load
Source: Sci Adv. 2019 Oct 9;5(10):eaay0244. doi: 10.1126/sciadv.aay0244 (PMC6785259; doi:10.1126/sciadv.aay0244)
Supplement: Download PDF [file aay0244_SM.pdf]

Science Advances
AAAS

advances.sciencemag.org/cgi/content/full/5/10/eaay0244/DC1

Supplementary Materials for

# Morphology and composition play distinct and complementary roles in the tolerance of plantar skin to mechanical load

Colin J. Boyle, Magdalena Plotczyk, Sergi Fayos Villalta, Sharad Patel, Shehan Hettiaratchy, Spyros D. Masouros, Marc A. Masen, Claire A. Higgins*

*Corresponding author. Email: c.higgins@imperial.ac.uk

Published 9 October 2019, *Sci. Adv.* **5**, eaay0244 (2019)
DOI: 10.1126/sciadv.aay0244

**This PDF file includes:**

Table S1. Antibodies used.
Table S2. Shear moduli based on rule-of-mixtures analysis.
Fig. S1. Histological analysis.
Fig. S2. Mechanical testing of skin.
Fig. S3. Constructing finite element models of the skin.

**Supplementary Materials**

**Table S1. Antibodies used.**

| Antibody Target | Company | Catalogue number | Dilution | Fixative |
|---|---|---|---|---|
| K9 | Progen | GPHK9-TY1 | 1 in 50 | 4% PFA |
| Laminin α3/Laminin-5 | R&D systems | MAB2144 | 1 in 25 | 4% PFA |
| Collagen 1 | Abcam | AB34710 | 1 in 200 | 4% PFA |
| DSG1 | Abcam | AB12077 | 1 in 50 | 4% PFA |
| Collagen IV | Abcam | AB6311 | 1 in 500 | 4% PFA |
| Loricrin | Biolegend | PRB-145P or 905101 | 1 in 200 | 4% PFA |

**Table S2. Shear moduli based on rule-of-mixtures analysis.** Tissue-level shear modulus is calculated by curve fitting compression and shear test data, while layer-level moduli are calculated using a Reuss model rule-of-mixtures approach.

| Layer | plantar (kPa) | non-plantar (kPa) |
|---|---|---|
| tissue-level | 4.86 | 1.19 |
| dermis | 2.55 | 1.06 |
| viable epidermis | 61.75 | 17.57 |
| stratum corneum | 86.76 | 42.28 |

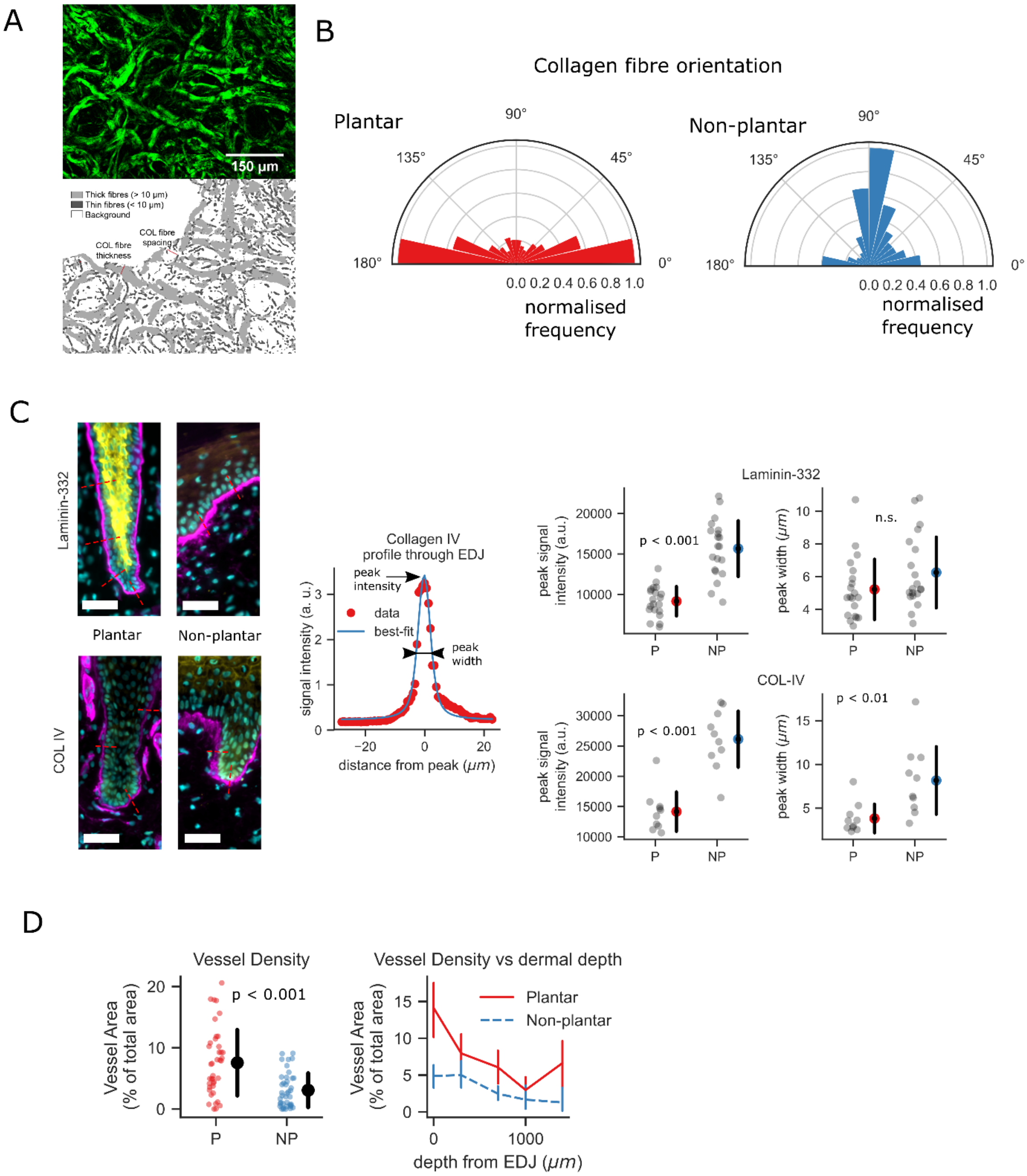


**Fig. S1. Histological analysis.** (**A**) Example of a segmented SHG image showing thick (grey) and thin (dark grey) collagen fibers. These images were then analysed using CurveAlign to extract the collagen thickness, orientation and alignment. (**B**) Orientation of collagen fibers in SHG images for non-plantar and plantar skin. Orientation was calculated using CurveAlign software. (**C**) The immunofluorescence signal across the epidermal-dermal junction (left) was measured by probing the data along trajectories traversing the EDJ (dashed red lines). The data along each trajectory was modelled as a Voigt distribution allowing the peak intensity and width to be measured robustly (centre). The intensity of Laminin-332 and Collagen IV was higher in non-plantar images (right). The thickness of the Collagen IV positive region was higher in non-plantar skin. (**D**) Images of collagen IV stained sections were analyzed to calculate the % area of positive collagen IV expression. This was taken as a measure of the density of vessels within the dermis. Plantar skin had a higher density of vessels, and this was particularly pronounced in the papillary dermis.

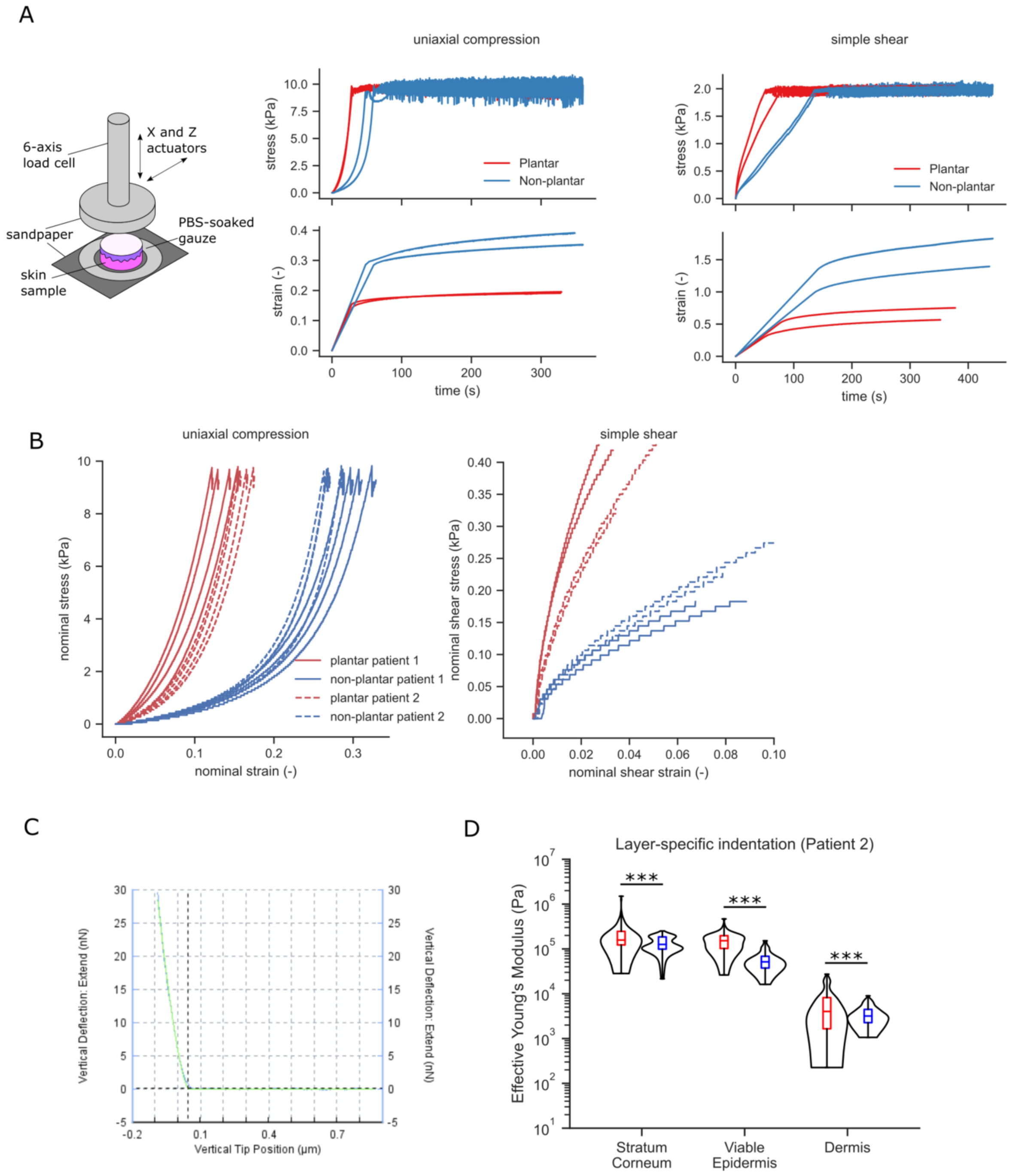


**Fig. S2. Mechanical testing of skin.** (**A**) Stress and strain data over time in uniaxial compression (left) and simple shear (right). (**B**) Example Stress-strain relations for both patients in uniaxial compression (left) and simple shear (right). (**C**) Example force-displacement curve from AFM indentation with a spherical indenter (example shown is viable epidermis) showing curve fit in green. (**D**) Layer-specific properties for a second patient. Student's two-sided t-test with unequal variances showed statistically significant differences between the skin types across all layers ($p<0.001$).

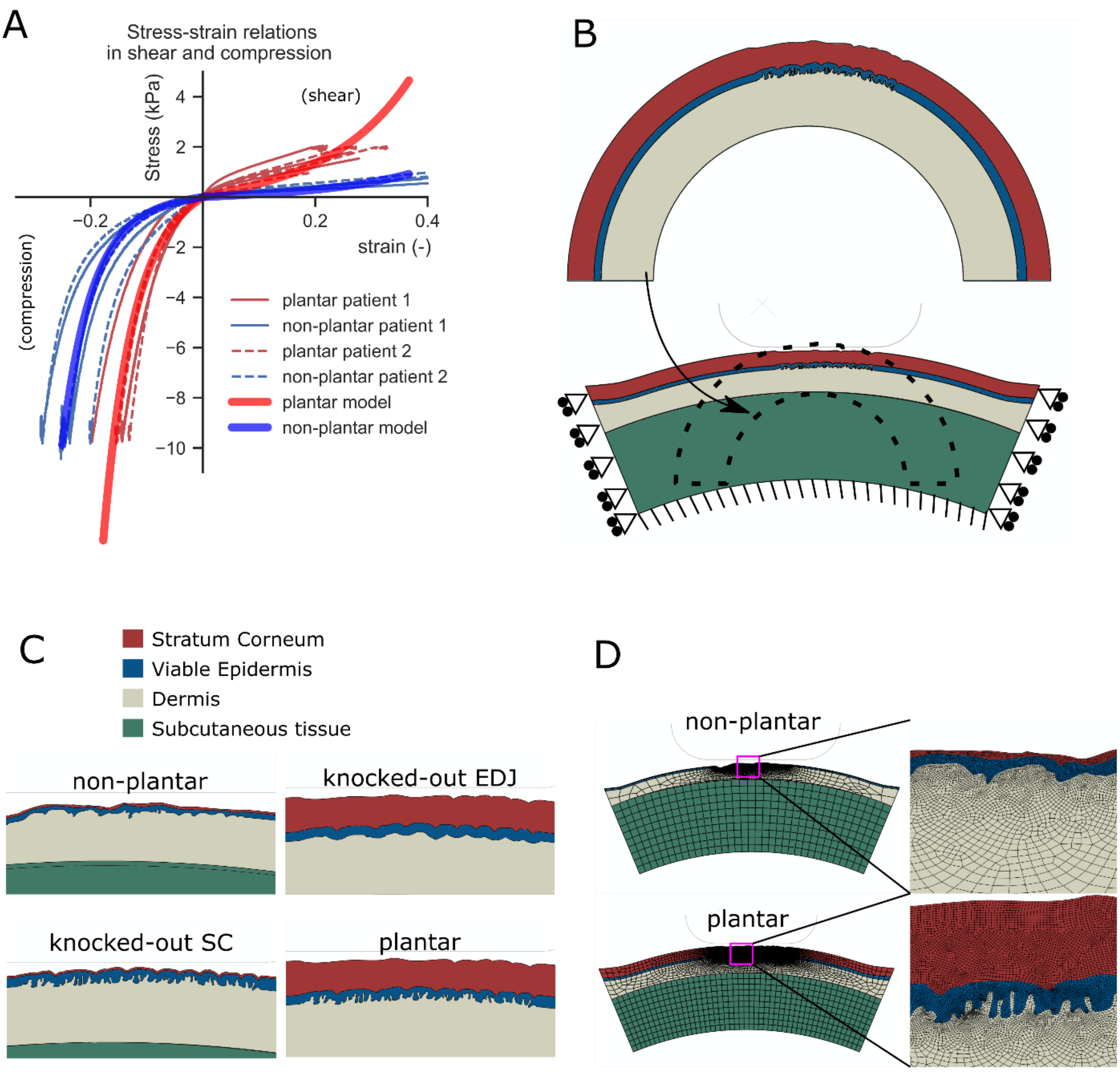


**Fig. S3. Constructing finite element models of the skin.** (**A**) The shear and compressive response of whole-skin samples were used to fit an Ogden hyperelastic model. (**B**) Skin biopsies tend to curl up due to the release of in-vivo tension. To create an *in vivo* model, simplified skin was added to the periphery of the histologically-derived geometry. This simplified skin was then deformed to reduce the curvature of the sample, thus creating a more in-vivo geometry. Subcutaneous tissue was inserted below the skin, which was attached to the lower surface of the dermis and was fixed at the bottom surface. Symmetry Boundary conditions were enforced at the left and right sides of the model. (**C**) Geometries representing non-plantar and plantar skin. Knock-out models in which either the stratum corneum or EDJ interdigitation were reduced to levels comparable to non-plantar skin. (**D**) The finite element meshes used to discretize the skin geometries. Higher mesh densities were used in the region of interest (insets), while a coarser mesh was used in peripheral tissue.
